# Solvability of a boundary value problem at resonance

**DOI:** 10.1186/s40064-016-3172-7

**Published:** 2016-09-07

**Authors:** A. Guezane-Lakoud, R. Khaldi, A. Kılıçman

**Affiliations:** 1 Laboratory of Advanced Materials, Faculty of Sciences, Badji Mokhtar-Annaba University, P.O. Box 12, 23000 Annaba, Algeria; 2 Department of Mathematics and Institute for Mathematical Research, Universiti Putra Malaysia (UPM), 43400 Serdang, Selangor Malaysia

**Keywords:** Fractional boundary value problem at resonance, Existence of solution, Schaefer fixed point theorem, Banach contraction principal, 34A08, 34B15

## Abstract

This paper concerns the solvability of a nonlinear fractional boundary value problem at resonance. By using fixed point theorems we prove that the perturbed problem has a solution, then by some ideas from analysis we show that the original problem is solvable. An example is given to illustrate the obatined results.

## Background

Boundary value problems (BVP) at resonance have been studied in many papers for ordinary differential equations (Feng and Webb 1997; Guezane-Lakoud and Frioui 2013; Guezane-Lakoud and Kılıçman 2014; Hu and Liu 2011; Jiang 2011; Kosmatov 2010, 2006; Mawhin 1972; Samko et al. 1993; Webb and Zima 2009; Zima and Drygas 2013), most of them considered the existence of solutions for the BVP at resonance making use of Mawhin coincidence degree theory (Liu and Zhao 2007). In Guezane-Lakoud and Kılıçman (2014), Han investigated the existence and multiplicity of positive solutions for the BVP at resonance by considering an equivalent non resonance perturbed problem with the same conditions. More precisely, he wrote the original problem $u^{\prime \prime }=f\left( t,u\right)$ as

$$u^{\prime \prime }+\beta ^{2}u=f\left( t,u\right) +\beta ^{2}u=g\left( t,u\right)$$

under the conditions $\beta \in \left( 0,\frac{\pi }{2}\right)$ and $f:[ 0,1] \times [ 0,\infty [ \rightarrow {\mathbb {R}}$ is continuous and $f\left( t,u\right) \ge -\beta ^{2}u.$ This result has been improved by Webb et al., in Samko et al. (1993) where the authors investigated a similar problem with various nonlocal boundary conditions.

In a recent study Mawhin (1972), Nieto investigated a resonance BVP by an other approach, that we will apply to a fractional boundary value problem to prove the existence of solutions.

The goal of this paper is to provide sufficient conditions that ensure the existence of solutions for the following fractional boundary value problem (P)

1 $$^{c}D_{0^{+}}^{q}u(t)=f\left( t,u(t),u^{\prime }(t)\right) ,\quad 0<t<1,$$

2 $$u\left( 0\right) =u^{\prime }(0)=0,\quad u^{\prime \prime }\left( 0\right) =2u\left( 1\right) ,$$

where $$f\in C\left( \left[ 0,1\right] \times {\mathbb {R}} \times {\mathbb {R}} ,{\mathbb {R}} \right)$$, $$2<q<3,$$$$^{c}D_{0^{+}}^{\alpha }$$ denotes the Caputo’s fractional derivative. The problem (P) is called at resonance in the sense that the associated linear homogeneous boundary value problem

$$^{c}D_{0^{+}}^{q}u(t)=0,u\left( 0\right) =u^{\prime }(0)=0,\quad u^{\prime \prime }\left( 0\right) =2u\left( 1\right),$$

has $$u(t)=ct^{2},$$$$c\in {\mathbb {R}}$$ as nontrivial solutions. In this case since Leray-Schauder continuation theory cannot be used, we will apply some ideas from analysis. Although these techniques have already been considered in Mawhin (1972) for ordinary differential equation but the present problem (P) is different since the nonlinearity *f* depends also on the derivative and the differential Eq. (1) is of fractional type.

Fractional boundary value problems at resonance have been investigated in many works such in Bai (2011), Han (2007), Infante and Zima (2008), where the authors applied Mawhin coincidence degree theory. Further for the existence of unbounded positive solutions of a fractional boundary value problem on the half line, see Guezane-Lakoud and Kılıçman (2014).

The organization of this work is as follows. In Sect. 2, we introduce some notations, definitions and lemmas that will be used later. Section 3 treats the existence and uniqueness of solution for the perturbed problem by using respectively Schaefer fixed point theorem and Banach contraction principal. Then by some analysis ideas, we prove that the problem (P) is solvable. Finally, we illustrate the obtained results by an example.

## Preliminaries

In this section, we present some Lemmas and Definitions from fractional calculus theory that can be found in Nieto (2013), Podlubny (1999).

### **Definition 1**

If $g\in C([a,b])$ and $\alpha >0,$ then the Riemann-Liouville fractional integral is defined by

$$I_{a^{+}}^{\alpha }g(t)=\frac{1}{\Gamma \left( \alpha \right) }\int _{a}^{t}\frac{g(s)}{(t-s)^{1-\alpha }}ds.$$

### **Definition 2**

Let $\alpha \ge 0,n=[\alpha ]+1.$ If $g\in C^{n}[a,b]$ then the Caputo fractional derivative of order $\alpha$ of *g* defined by

$$^{c}D_{a^{+}}^{\alpha }g(t)=\frac{1}{\Gamma \left( n-\alpha \right) }\int _{a}^{t}\frac{g^{\left( n\right) }(s)}{(t-s)^{\alpha -n+1}}ds,$$

exists almost everywhere on [*a*, *b*] $([\alpha ]$ is the integer part of $\alpha ).$

### **Lemma 3**

*For *$$\alpha >0,$$$$g\in C(\left[ 0,1\right] ,{\mathbb {R}} ),$$*the homogenous fractional differential equation *

$$^{c}D_{a^{+}}^{\alpha }g(t)=0$$

*has a solution *

$$g(t)=c_{0}+c_{1}t+c_{2}t^{2}+{\cdots}+c_{n-1}t^{n-1},$$

*where*, $$c_{i}\in {\mathbb {R}} ,$$$$i=0,{\ldots},n-1,$$*herenis the smallest integer greater than or equal to*$$\alpha .$$

### **Lemma 4**

*Let*$$p,q\ge 0,$$$$f\in L_{1}\left[ a,b\right] .$$*Then*$$I_{0^{+}}^{p}I_{0^{+}}^{q}f\left( t\right) =I_{0^{+}}^{p+q}f\left( t\right) =I_{0^{+}}^{q}I_{0^{+}}^{p}f\left( t\right)$$*and*$$^{c}D_{0^{+}}^{q}I_{0^{+}}^{q}f\left( t\right) =f\left( t\right) ,$$*for all*$$t\in \left[ a,b\right]$$.

Now we start by solving an auxiliary problem.

### **Lemma 5**

*Let*$2<q<3$*and*$y\in C\left[ 0,1\right] .$*The linear fractional boundary value problem*

3 $$\left\{\begin{array}{ll} ^{c}D_{0^{+}}^{q}u\left( t\right) =y(t). \\ u\left( 0\right) =u^{\prime }(0)=0,\quad u^{\prime \prime }\left( 0\right) =2u(1),\end{array}\right.$$

*has a solution if and only if*$I_{0^{+}}^{q}y(1)=0,$*in this case the solution can be written as*

4 $$u(t)-t^{2}u(1)=\frac{1}{\Gamma \left( q\right) }\int _{0}^{1}H(t,s)y(s)ds,$$

*where*

5 $$H(t,s)=\left\{ \begin{array}{ll} (t-s)^{q-1}+t^{2}(1-s)^{q-1},\quad \ s\le t, \\ t^{2}(1-s)^{q-1},\quad t\le s.\end{array}\right.$$

### *Proof*

Applying Lemma 3 to (3) we get

6 $$u\left( t\right) =I_{0^{+}}^{q}y\left( t\right) +c_{0}+c_{1}t+c_{2}t^{2}.$$

Differentiating both sides of (6), it yields

7 $${u^\prime }(t) = I_{{0^ + }}^{q - 1}y\left( t \right) + {c_1} + 2{c_2}t,$$

8 $${u^{\prime \prime }}\left( t \right) = I_{{0^ + }}^{q - 2}y\left( t \right) + 2{c_2}.$$

The first condition in (3) gives $c_{0}=c_{1}=0,$ the second one implies that $I_{0^{+}}^{q}y(1)=0,$ hence (3) has solution if and only if $I_{0^{+}}^{q}y(1)=0$, then the problem (3) has an infinity of solutions given by

9 $$u(t)=I_{0^{+}}^{q}y(t)+c_2t^{2}.$$

Now we try to rewrite the function *u*. We have

$$u(1)-u^{\prime \prime }(0)=-I_{0^{+}}^{q}y(1)+c$$

then

$$c=I_{0^{+}}^{q}y(1)+u(1)$$

substituting *c* by its value in (9) we obtain

$$\begin{aligned} u(t)&= I_{0^{+}}^{q}y(t)+t^{2}I_{0^{+}}^{q}y(1)+t^{2}u(1) \\&= \frac{1}{\Gamma \left( q\right) }\int _{0}^{1}H(t,s)y(s)ds+t^{2}u(1), \end{aligned}$$

Hence the linear problem can be written as

$$u(t)-t^{2}u(1)=\frac{1}{\Gamma \left( q\right) }\int _{0}^{1}H(t,s)y(s)ds,$$

where $$H(t,s)=\left\{ \begin{array}{ll} (t-s)^{q-1}+t^{2}(1-s)^{q-1},\quad \ s\le t, \\ t^{2}(1-s)^{q-1},\quad t\le s.\end{array}\right.$$ The kernel *H*(*t*, *s*) is continuous according to both variables *s*, *t* on $\left[ 0,1\right] \times \left[ 0,1\right]$ and is positive. $\square$

Consequently the nonlinear problem (1) is transformed to the integral equation

10 $$u(t)-t^{2}u(1)=\frac{1}{\Gamma \left( q\right) }\int _{0}^{1}H(t,s)f(s,u(s),u^{\prime }(s))ds.$$

Define a new function $v(t)=u(t)-t^{2}u(1)$. To find a solution *u* we have to find *v* and *u*(1). Note $v_{c}(t)=u(t)-t^{2}c,$ we try to solve for every $v_{c}$ the problem

11 $$v_{c}(t)=\frac{1}{\Gamma \left( q\right) }\int _{0}^{1}H(t,s)f(s,v_{c}(s)+cs^{2},v_{c}^{\prime }(s)+2cs)ds,$$

if $v_{c}$ is a solution of (11) with $c=u(1)$ then *u* is a solution of (1).

## Existence and uniqueness results

Let *E* be the Banach space of all functions $u\in C^{1}\left[ 0,1\right]$ into ${\mathbb {R}} ,$ equipped with the norm $\left\| u\right\| =\max \left( \left\| u\right\| _{\infty },\left\| u^{\prime }\right\| _{\infty }\right)$ where $\left\| u\right\| _{\infty }=\max _{t\in \left[ 0,1\right] }\left| u\left( t\right) \right|$. Denote by $L^{1}\left( \left[ 0,1\right] ,{\mathbb {R}} \right)$ the Banach space of Lebesgue integrable functions from $\left[ 0,1\right]$ into ${\mathbb {R}}$ with the norm $\left\| y\right\| _{L^{1}}=\int _{0}^{1}\left| y\left( t\right) \right| dt.$ Define the integral operator $T:E\rightarrow E$ by

12 $$Tu(t)=t^{2}u(1)+\frac{1}{\Gamma \left( q\right) }\int _{0}^{1}H\left( t,s\right) f\left( s,u\left( s\right) ,u^{\prime }\left( s\right) \right) ds,$$

and the corresponding perturbed operator $T_{c}:E\rightarrow E$ by

13 $$T_{c}v(t)=\frac{1}{\Gamma \left( q\right) }\int _{0}^{1}H\left( t,s\right) f\left( s,v\left( s\right) +cs^{2},v^{\prime }\left( s\right) +2cs\right) ds.$$

### **Theorem 1**

*Assume that there exist nonnegative functions g*, *h*, *k*$$\in$$$$L^{1}\left( \left[ 0,1\right] ,{\mathbb {R}} _{+}^{*}\right)$$* and *$$0\le \alpha <1$$*such that*

14 $$\left| f(t,x,\overline{x})\right| \le k(t)\left| x\right| ^{\alpha }+h(t)\left| \overline{x}\right| ^{\alpha }+g(t),\text { }\forall (t,x,\overline{x})\in \left[ 0,1\right] \times {\mathbb {R}} ^{2},$$

15 $$\Gamma \left( q\right) -\left( q+1\right) \left( \left\| k\right\| _{L^{1}}+\left\| h\right\| _{L^{1}}\right) >0.$$

*Then the map *$T_{c}$*has at least one fixed point *$v^{*}\in E.$

We apply Schaefer fixed point theorem to prove Theorem 1.

### **Theorem 2**

*LetAbe a completely continuous mapping of a Banach spaceXinto it self, such that the set*$\left\{ x\in X:x=\lambda Ax,0<\lambda <1\right\}$*is bounded, thenAhas a fixed point.*

### Proof of Theorem 1

By Arzela-Ascoli Theorem we can easly show that $T_{c}$ is a completely continuous mapping.

Now, let us prove that the set $\left\{ v\in E:v=\lambda T_{c}v,0<\lambda <1\right\}$ is bounded. Endeed for $\lambda \in \left( 0,1\right)$ such that $v=\lambda T_{c}(v),$ we have

$$v(t)=\frac{\lambda }{\Gamma \left( q\right) }\int _{0}^{1}H(t,s)f(s,v(s)+cs^{2},v^{\prime }(s)+2cs)ds,$$

remarking that *H*(*t*, *s*) is continuous according to both variables *s*, *t* on $\left[ 0,1\right] \times \left[ 0,1\right]$, nonnegative and $0\le H(t,s)\le 2$ then using assumptions (14) and (15), we get

$$\begin{aligned} \left| v(t)\right|&\le \frac{2\lambda }{\Gamma \left( q\right) }\int _{0}^{1}\left[ k(s)\left| v(s)+cs^{2}\right| ^{\alpha }+h(s)\left| v^{\prime }(s)+2cs\right| ^{\alpha }+g(s)\right] ds \\&\le \frac{2}{\Gamma \left( q\right) }\left[ \left\| k\right\| _{L^{1}}\left( \left\| v\right\| _{\infty }+\left| c\right| \right) ^{\alpha }+\left\| h\right\| _{L^{1}}\left( \left\| v^{\prime }\right\| _{\infty }+2\left| c\right| \right) ^{\alpha }+\left\| g\right\| _{L^{1}}\right] \\&\le \frac{2\max \left( \left\| k\right\| _{L^{1}},\left\| h\right\| _{L^{1}}\right) }{\Gamma \left( q\right) }\left( \left\| v\right\| +2\left| c\right| \right) ^{\alpha }+\frac{2}{\Gamma \left( q\right) }\left\| g\right\| _{L^{1}}, \end{aligned}$$

thus,

16 $$\begin{aligned} \left\| v\right\| _{\infty }\le \frac{2\max \left( \left\| k\right\| _{L^{1}},\left\| h\right\| _{L^{1}}\right) }{\Gamma \left( q\right) }\left( \left\| v\right\| +2\left| c\right| \right) ^{\alpha }+\frac{2}{\Gamma \left( q\right) }\left\| g\right\| _{L^{1}}. \end{aligned}$$

Let $$H^{\prime }(t,s)=H_{t}(t,s)=\left\{ \begin{array}{ll} \left( q-1\right) (t-s)^{q-2}+2t(1-s)^{q-1},\text { \ }s\le t, \\ 2t(1-s)^{q-1},\quad t\le s.\end{array}\right.,$$ then $H_{t}(t,s)$ is continuous according to both variables *s*, *t* on $\left[ 0,1\right] \times \left[ 0,1\right]$, nonnegative and $0\le H_{t}(t,s)\le q+1$. We have

$$v^{\prime }(t)=\frac{\lambda }{\Gamma \left( q\right) }\int _{0}^{1}H_{t}(t,s)f(s,v(s)+cs^{2},v^{\prime }(s)+2cs)ds.$$

Similarly we get

17 $$\begin{aligned} \left\| v^{\prime }\right\| _{\infty }\le \frac{\left( q+1\right) \max \left( \left\| k\right\| _{L^{1}},\left\| h\right\| _{L^{1}}\right) }{\Gamma \left( q\right) }\left( \left\| v\right\| +2\left| c\right| \right) ^{\alpha }+\frac{q+1}{\Gamma \left( q\right) }\left\| g\right\| _{L^{1}}. \end{aligned}$$

From (16) and (17) it yields

18 $$\begin{aligned} \left\| v\right\| \le \frac{\left( q+1\right) \max \left( \left\| k\right\| _{L^{1}},\left\| h\right\| _{L^{1}}\right) }{\Gamma \left( q\right) }\left( \left\| v\right\| +2\left| c\right| \right) ^{\alpha }+\frac{q+1}{\Gamma \left( q\right) }\left\| g\right\| _{L^{1}}. \end{aligned}$$

From here one can get

$$\begin{aligned} \left\| v\right\| \le \frac{\Gamma \left( q\right) }{\Gamma \left( q\right) -\left( q+1\right) \max \left( \left\| k\right\| _{L^{1}},\left\| h\right\| _{L^{1}}\right) }\left( 2\left| c\right| +1+\frac{q+1}{\Gamma \left( q\right) }\left\| g\right\| _{L^{1}}\right) , \end{aligned}$$

we conclude that *v* is bounded independently of $\lambda$, then Schaefer fixed point theorem implies $T_{c}$ has at least a fixed point. Hence equation

19 $$v(t)=\frac{1}{\Gamma \left( q\right) }\int _{0}^{1}H\left( t,s\right) f\left( s,v\left( s\right) +cs^{2},v^{\prime }\left( s\right) +2cs\right) ds.$$

has at least one solution in *E*. The proof is complete. $\square$

The uniqueness result is given by the following Theorem:

### **Theorem 3**

*Assume there exist nonnegative functions *$$g,h\in L^{1}\left( \left[ 0,1\right] ,{\mathbb {R}} _{+}\right)$$* such that for all *$$x,y,\overline{x},\overline{y}\in {\mathbb {R}} ,$$$$t\in \left[ 0,1\right]$$*one has*

20 $$\begin{aligned}&\left| f(t,x,\overline{x})-f(t,y,\overline{y})\right| \le g(t)\left| x-y\right| +h(t)\left| \overline{x}-\overline{y}\right| , \end{aligned}$$

21 $$\begin{aligned}&\Gamma \left( q\right) -\left( q+1\right) \left( \left\| g\right\| _{L^{1}}+\left\| h\right\| _{L^{1}}\right) >0. \end{aligned}$$

*Then *$T_{c}$*has a unique fixed point *$v_{c}^{*}$*in E*.

### Proof

Let *v* and $w\in E$, then by (20) we get

$$\begin{aligned}&\left| T_{c}v(t)-T_{c}w(t)\right| \le \frac{1}{\Gamma \left( q\right) }\int _{0}^{1}H(t,s) \\&\times \quad \left| f(s,v(s)+cs^{2},v^{\prime }(s)+2cs)-f(s,w(s)+cs^{2},w^{\prime }(s)+2cs)\right| ds \\&\quad \le \frac{1}{\Gamma \left( q\right) }\int _{0}^{1}H(t,s)\left( g(s)\left| v(s)-w(s)\right| +h(s)\left| v^{\prime }(s)-w^{\prime }(s)\right| \right) ds \\&\quad \le \frac{2\left\| v-w\right\| \left( \left\| g\right\| _{L^{1}}+\left\| h\right\| _{L^{1}}\right) }{\Gamma \left( q\right) }, \end{aligned}$$

thus

22 $$\begin{aligned} \left\| T_{c}v-T_{c}w\right\| _{\infty }\le \frac{2\left( \left\| g\right\| _{L^{1}}+\left\| h\right\| _{L^{1}}\right) }{\Gamma \left( q\right) }\left\| v-w\right\| . \end{aligned}$$

Similarly we get

23 $$\begin{aligned} \left\| T_{c}^{\prime }v-T_{c}^{\prime }w\right\| _{\infty }\le \frac{\left( q+1\right) \left( \left\| g\right\| _{L^{1}}+\left\| h\right\| _{L^{1}}\right) }{\Gamma \left( q\right) }\left\| v-w\right\| , \end{aligned}$$

consequently

$$\begin{aligned} \left\| T_{c}v-T_{c}w\right\|\le \,& \frac{\left( q+1\right) \left( \left\| g\right\| _{L^{1}}+\left\| h\right\| _{L^{1}}\right) }{\Gamma \left( q\right) }\left\| v-w\right\| \\\le \,& l\left\| v-w\right\| , \end{aligned}$$

where $l=\frac{\left( q+1\right) \left( \left\| g\right\| _{L^{1}}+\left\| h\right\| _{L^{1}}\right) }{\Gamma \left( q\right) }.$ The assumption (21) implies that $l<1$, so the Banach contraction principle ensure the uniqueness of the fixed point. The proof is complete. $\square$

Let us remark that under the assumptions of Theorem 3, the map $$\Psi :{\mathbb {R}}\rightarrow E,$$$$\Psi \left( c\right) =v_{c}^{*}$$ is continuous. Moreover the map $$\Lambda :{\mathbb {R}}\rightarrow {\mathbb {R}},$$$$\Lambda =\Phi \circ \Psi ,\Lambda \left( c\right) =v_{c}^{*}\left( 1\right)$$ is also continuous, where $$\Phi :E\rightarrow {\mathbb {R}},$$$$\Phi \left( v\right) =v\left( 1\right)$$ and $$v_{c}^{*}$$ is the unique fixed point of $$T_{c}$$.

Let us show that the problem (1–2) is solvable.

### **Theorem 4**

*Under the assumptions of Theorems* 1*and*  3*and if*

$$\begin{aligned} \lim _{\left( u,v\right) \rightarrow \pm \infty }f\left( t,u,v\right) =\pm \infty \end{aligned}$$

*uniformly on*$\left[ 0.1\right]$, *then the problem *(1–2) *has at least one solution inE*. $(\left( u,v\right) \rightarrow +\infty ,$*ie*. $u\rightarrow +\infty$*and*$v\rightarrow +\infty ).$

### *Proof*

The condition $\lim _{\left( u,v\right) \rightarrow \pm \infty }f\left( t,u,v\right) =\pm \infty$ is assumed to avoid the case $f(t,u(t),u^{\prime }(t))=y\left( t\right)$ where the problem may have no solution (in the case $I_{0^{+}}^{q}y(1)\ne 0).$ If we prove that $\lim _{c\rightarrow \pm \infty }\Lambda \left( c\right) =\pm \infty ,$ then there exists $c^{*}\in {\mathbb {R}}$ such that $\Lambda \left( c^{*}\right) =0$ consequently $c^{*}=u_{c^{*}}(1)$ hence $u_{c^{*}}(t)=v_{c^{*}}^{*}(t)+t^{2}c^{*}$ is a solution of the nonlinear problem (1–2).

Now taking into account (18) we get $\lim _{c\rightarrow +\infty }\frac{\left\| v_{c}^{*}\right\| }{c}=0.$ Since the norms of $\left( v_{c}^{*}(s)+cs^{2}\right)$ and $\left( v_{c}^{*\prime }(s)+2cs\right)$ growth asymptotically as *c*,  $H\left( t,s\right)$ is nonnegative and continuous and $\lim _{\left( u,v\right) \rightarrow \pm \infty }f\left( t,u,v\right) =\pm \infty ,$ then from (19) it yields $\lim _{c\rightarrow \pm \infty }\Lambda \left( c\right) =\pm \infty .$ The proof is complete. $\square$

### *Example 5*

The following fractional boundary value problem

24 $$\begin{aligned} \left\{\begin{array}{ll} ^{c}D_{0^{+}}^{\frac{5}{2}}u(t)=\frac{\left( 1+t^{2}\right) }{8}\left( \frac{u^{\frac{7}{3}}}{1+u^{2}}+\frac{\left( u^{\prime }\right) ^{\frac{7}{3}}}{1+\left( u^{\prime }\right) ^{2}}\right) +\left( 1+t\right) ^{2},\quad 0<t<1, \\ u\left( 0\right) =u^{\prime }(0)=0,\quad u^{\prime \prime }\left( 0\right) =2u\left( 1\right) ,\end{array}\right. \end{aligned}$$

is solvable in *E*.

### Proof

We have $q=\frac{5}{2}$ and

$$\begin{aligned} \left| f(t,x,\overline{x})\right|&= \left| \frac{\left( 1+t^{2}\right) }{8}\left( \frac{x^{\frac{7}{3}}}{1+x^{2}}+\frac{\overline{x}^{\frac{7}{3}}}{1+\overline{x}^{2}}\right) +\left( 1+t\right) ^{2}\right| \\&\le \frac{\left( 1+t^{2}\right) }{8}\left| x\right| ^{\frac{1}{3}}+\frac{\left( 1+t^{2}\right) }{8}\left| \overline{x}\right| ^{\frac{1}{3}}+\left( 1+t\right) ^{2} \\&\le k(t)\left| x\right| ^{\frac{1}{3}}+h(t)\left| \overline{x}\right| ^{\frac{1}{3}}+g(t), \end{aligned}$$

where

$$\begin{aligned} k(t)=h(t)=\frac{\left( 1+t^{2}\right) }{8},\quad g(t)=\left( 1+t\right) ^{2},\quad \left\| k\right\| _{L^{1}}=\frac{1}{6}, \end{aligned}$$

some calculus give

$$\begin{aligned} \Gamma \left( q\right) -\left( q+1\right) \left( \left\| k\right\| _{L^{1}}+\left\| h\right\| _{L^{1}}\right) =0.16267>0. \end{aligned}$$

Applying Theorem 1 we conclude that the map $T_{c}$ has at least one fixed point $v^{*}\in E$. Now we have

$$\begin{aligned}&\left| f(t,x,\overline{x})-f(t,y,\overline{y})\right| \\&\quad \le \frac{\left( 1+t^{2}\right) }{8}\left| \frac{x^{\frac{7}{3}}}{1+x^{2}}-\frac{y^{\frac{7}{3}}}{1+y^{2}}\right| +\frac{\left( 1+t^{2}\right) }{8}\left| \frac{\overline{x}^{\frac{7}{3}}}{1+\overline{x}^{2}}-\frac{\bar{y}^{\frac{7}{3}}}{1+\bar{y}^{2}}\right| \\&\quad \le \left( 0.8\right) \frac{\left( 1+t^{2}\right) }{8}\left| x-y\right| +\left( 0.8\right) \frac{\left( 1+t^{2}\right) }{8}\left| \overline{x}-\overline{y}\right| \\&\quad =G(t)\left| x-y\right| +H(t)\left| \overline{x}-\overline{y}\right| , \end{aligned}$$

where $G(t)=H(t)=\left( 0.1\right) \left( 1+t^{2}\right) ,$ hence we get

$$\begin{aligned} \Gamma \left( q\right) -\left( q+1\right) \left( \left\| G\right\| _{L^{1}}+\left\| H\right\| _{L^{1}}\right) =0.396\,01>0. \end{aligned}$$

In view of Theorem 3, $T_{c}$ has a unique fixed point $v_{c}^{*}$ in *E*. It is easy to see that

$$\begin{aligned} \lim _{\left( u,v\right) \rightarrow \pm \infty }f\left( t,u,v\right) = \lim _{\left( u,v\right) \rightarrow \pm \infty }\left[ \frac{\left( 1+t^{2}\right) }{8}\left( \frac{u^{\frac{7}{3}}}{1+u^{2}}+\frac{v^{\frac{7}{3}}}{1+v^{2}}\right) +\left( 1+t\right) ^{2}\right] =\pm \infty . \end{aligned}$$

From the above discussion and Theorem 4 we conclude that the problem (24) is solvable in *E*. $\square$

## Conclusion

The goal of this paper was to provide sufficient conditions in order to ensure the existence of solutions for the following fractional boundary value problem

$$\begin{aligned} ^{c}D_{0^{+}}^{q}u(t)&= f\left( t,u(t),u^{\prime }(t)\right) ,\quad 0<t<1, \\ u\left( 0\right)&= u^{\prime }(0)=0,\quad u^{\prime \prime }\left( 0\right) =2u\left( 1\right) , \end{aligned}$$

where $$f\in C\left( \left[ 0,1\right] \times {\mathbb {R}} \times {\mathbb {R}} ,{\mathbb {R}} \right)$$, $$2<q<3,$$$$^{c}D_{0^{+}}^{\alpha }$$ denotes the Caputo’s fractional derivative. By using fixed point theorems we proved that the perturbed problem has a solution, then we also show that the original problem is solvable. An example is provided n order to illustrate the results.
